# Activation of blood coagulation and thrombin generation in acute ischemic stroke treated with rtPA

**DOI:** 10.1007/s11239-017-1544-7

**Published:** 2017-09-06

**Authors:** Sarah Goldman, Shannon M. Prior, Jan P. Bembenek, Maciej Niewada, Elżbieta Broniatowska, Anna Członkowska, Saulius Butenas, Anetta Undas

**Affiliations:** 1 Krakow Center for Medical Research and Technology, John Paul II Hospital, Krakow, Poland; 2 Department of Biochemistry, University of Vermont, Colchester, VT USA; 3 2nd Department of Neurology, Institute of Psychiatry and Neurology, Warsaw, Poland; 4 Department of Bioinformatics and Telemedicine, Jagiellonian University Medical College, Krakow, Poland; 5 Institute of Cardiology, Jagiellonian University Medical College, 80 Pradnicka St., 31-202 Krakow, Poland

**Keywords:** Coagulation factor, Stroke, Thrombolysis, Thrombin generation

## Abstract

The impact of thrombolysis with recombinant tissue plasminogen activator (rtPA) on blood coagulation in acute ischemic stroke (AIS) patients is not completely understood. We studied the effect of thrombolysis on the thrombin generation (TG) profile as well as coagulant activity of activated factors IX (FIXa), XI (FXIa) and tissue factor (TF) in AIS patients. In a case-control study, TG parameters as well as FIXa, FXIa and TF levels were assessed in 95 AIS patients, including individuals receiving rtPA treatment within 4.5 h since AIS onset (n = 71, 74.7%) and those ineligible for thrombolysis (n = 24, 25.3%). Blood samples were collected at baseline and after 24 h since admission. The two groups were similar with regard to demographics and clinical factors. In thrombolysed patients, all TG parameters measured after 24 h were markedly decreased, with strongest impact on lag time (LT), when compared with the baseline values (81.3% longer LT, p < 0.0001), as well as when compared to the non-thrombolysed group (86% longer LT, p = 0.002). In non-thrombolysed AIS patients the TG remained unaltered. Logistic regression adjusted for potential confounders showed that high baseline ETP value (the top quartile) was solely predicted by the presence of circulating FIXa, whereas after 24 h FXIa predicted high ETP in the subgroup of thrombolysed and in all AIS patients. Thrombolysis in AIS patients markedly attenuates the TG. Elevated FXIa contributes to thrombin formation capacity after 24 h, highlighting a role of this factor in the regulation of blood coagulation in AIS.

**Electronic supplementary material:**

The online version of this article (doi:10.1007/s11239-017-1544-7) contains supplementary material, which is available to authorized users.

## Introduction

Stroke is one of the leading cause of death in adults in the United States [1]. Up to 85% of strokes are ischemic [2].

Several studies have addressed the issue of thrombin generation (TG) in acute ischemic stroke (AIS). TG is enhanced in patients with AIS [3]. Carcaillon et al. showed that high endogenous thrombin potential (ETP) and peak thrombin concentration were positively associated with the AIS risk [4].

Little is known about the impact of the thrombolytic therapy on TG in AIS. Tanne et al. reported that in thrombolysed patients 2 h following AIS, plasma thrombin-antithrombin complexes (TAT) increased by 360%, and then fell after 24 h to baseline values, whereas in nontreated patients it remained unchanged [5]. Balogun et al. studied the TG parameters in rtPA treated and nontreated patients with AIS using calibrated automated thrombography (CAT) within 48 h since the event and 2 weeks thereafter, and no intergroup differences in the TG profiles were observed [6].

It has been reported that patients with elevated plasma active tissue factor (TF) levels have a greater risk of ischemic stroke [7]. The presence of TF and activated factor XI (FXIa) may predispose patients with atrial fibrillation to AIS [8], and is associated with worse clinical outcome [9]. To our knowledge, active TF, activated factor FIX (FIXa) and FXIa have not yet been studied in thrombolysed AIS patients.

## Aims

We sought to determine the impact of thrombolytic therapy on the TG profile as well as coagulant activity of FIXa, FXIa and TF in AIS patients.

## Materials and methods

Consecutive patients (≥18 years old) admitted to the 2nd Department of Neurology at the Institute of Psychiatry and Neurology in Warsaw, Poland, with signs and symptoms of AIS were recruited from January to December 2014. The AIS diagnosis was made based on clinical symptoms according to the World Health Organization definition [10] and brain imaging.

Exclusion criteria were as follows: hemorrhagic stroke, subarachnoid hemorrhage, oral anticoagulation therapy with vitamin K antagonists [i.e. International Normalized Ratio (INR) >1.7 at the enrolment] or non-vitamin K antagonist oral anticoagulants (if taken within the previous 24 h), end-stage renal disease, history of myocardial infarction or venous thromboembolism within the previous 3 months, known hemorrhagic diathesis, malignancy or severe infection. The two prespecified subgroups represented individuals who received rtPA within 4.5 h after stroke onset according to the current guidelines, and those who were not eligible for this treatment, but admitted within 24 h since stroke onset [11], including hypertensive patients, whose blood pressure could not be lowered to systolic ≤185 mmHg or diastolic ≤110 mmHg and those admitted >4.5 h (following the guidelines [11]) since stroke onset. Aspirin was introduced after 24–36 h since rtPA treatment. No one received heparins or oral anticoagulants within the first 24 h since admission.

Neurological deficit severity was assessed on admission and 24 h thereafter with National Institutes of Health Stroke Scale (NIHSS) [12] by physicians, who were blinded to coagulation tests. All participants gave their written informed consent, and the study was approved by the Ethical Committee.

### Laboratory investigations

Blood samples were drawn from an antecubital vein with minimal stasis on admission (before treatment) and after 24 h. Blood cell count, glucose, creatinine, lipid profile, C-reactive protein (CRP), Activated Partial Thromboplastin Time (APTT) and INR were measured using routine techniques. Fibrinogen was determined using the Clauss assay.

### Thrombin generation assay

To assess the TG profile we used the assay previously described in details [13]. Citrated plasma samples were thawed at 37 °C for 3 min and 5 mg/mL corn trypsin inhibitor (CTI) was immediately added to achieve a 0.1 mg/mL final concentration. 80 µL of each plasma sample was added to a 96-well plate and relipidated TF at a final 5 pM concentration was added. 20 µL of a 2.5 mM Z-GGR-AMC/90 mM CaCl_2_ solution in Hepes-buffered saline (HBS) was added to plasma samples to achieve final concentrations of 417 µM/15 mM, respectively, followed by a 3 min incubation period at 37 °C to allow recalcification of the plasma. 20 µL of a 120 µM phospholipid vesicles (PCPS) solution in HBS was then added to plasma samples to achieve a final concentration of 20 µM, thus initiating TG. Fluorescence readings began immediately and hydrolysis of the AMC (7-amino-4-methylcoumarin) substrate (at 370 nm excitation and 460 nm emission wavelengths) was followed over a 3600s period. Changes in fluorescence were converted to thrombin concentration using a calibration curve built by sequential dilutions of human thrombin.

Pooled citrate platelet-poor plasma (PPP) was prepared in-house using 10 healthy donors [14]. CTI, a contact pathway inhibitor of coagulation, was prepared as described [15, 16]. PCPS composed of 25% dioleoyl-*sn*-glycero-3-phospho-l-serine and 75% 1,2-dioleoyl-*sn*-glycero-3-phosphocholine (both from Avanti Polar Lipids, Inc; Alabaster, AL) were prepared as described [17]. TF was relipidated as described previously [15]. The fluorogenic substrate used was benzyloxycarbonyl-Gly-Gly-Arg-7-amido-4methylcoumarin· HCl (Z-GGR-AMC) (Bachem, Torrance, CA). Human thrombin was produced in-house [18]. HBS buffer was prepared using 20 mM *N*-[2-hydroxyethyl]piperazine-NN-[2-ethanesulfonic acid] (HEPES) and 0.15 M NaCl, pH 7.4 (Fisher Scientific, Waltham, MA). The plate reader used was the BioTek Synergy 4 and analysis was performed using the Gen5 plate reader software (BioTek, Winooski, VT).

### Tissue and coagulation factors

Plasma was thawed at 37 °C in the presence of 0.1 mg/mL CTI (prepared as previously described) [15]. CaCl_2_ to a final 15 mM concentration was added, and the plasma incubated for 1 min; clotting was initiated by the addition of 2 μM PCPS composed of 25% dioleoyl-sn-glycero-3-phospho-l-serine and 75% of 1,2-dioleoyl-sn-glycero-3-phosphocholine (both from Avanti Polar Lipids, Inc; Alabaster, AL, USA) and prepared as described previously [17]. In parallel, inhibitory monoclonal anti-FXI (αFXI-2), anti-FIX (αFIX-91) or anti-TF (αTF-5) antibodies (both produced in house) at a final 0.1 mg/mL concentration were individually added to the same plasma prior to CaCl_2_ addition. αFXI-2 is specific for FXI/XIa and inhibits FIX activation by FXIa [19]. αTF-5 binds specifically to TF and interferes with TF/FVIIa complex formation [20]. Clotting times were determined using the ST8 instrument (Diagnostica Stago, Parsippany, NJ, USA). FXIa, FIXa and TF activity in plasma was calculated from calibration curves developed with human FIXa or FXIa (gifts from Dr. R. Jenny from Haematologic Technologies, Inc., Essex Junction, VT, USA) or relipidated [15] TF1-243 (a gift from Dr. R. Lundblad from Baxter Healthcare Corp., Duarte, CA, USA) in pooled 10-donor normal plasma. The detectability limit for TF was 0.1 pM, for FXIa 0.25 pM, and for FIXa 30 pM.

### Statistical analysis

Continuous variables were checked for normal distribution with the Shapiro–Wilk test. Data are expressed as mean ± standard deviation or median (interquartile range). To assess the differences between pre- and post-treatment values of continuous variables, paired Student *t* test, or the Wilcoxon signed-ranks test were applied. For nominal variables the McNemar’s test was used. Categorical variables were compared by χ^2^ test or Fisher’s exact test. The Pearson’s correlation coefficient or Spearman’s rank correlation coefficient were calculated to assess the linear correlations between variables with a normal or non-normal distribution, respectively. Multivariable logistic regression final models were adjusted for age, sex, BMI and fibrinogen levels. Two-sided p values of <0.05 were considered statistically significant. Analysis was performed using STATISTICA 12.0 software package (Stat Soft Inc., Tulsa, USA, 2011).

## Results

We studied 95 patients with AIS, including 71 (74.7%) treated with rtPA. Twenty-four subjects (25.3%) were unsuitable for thrombolysis and served as the control patients. Demographic and clinical parameters were similar in both groups (Table 1), however, the studied groups differed with regard to blood pressure on admission. Time from stroke onset to hospital admission was shorter in rtPA treated patients [2.0 (1.6–2.6) vs 10.1 (7.0–20.3) h, p < 0.0001]. NIHSS at baseline correlated inversely with the time from stroke onset to admission (r = −0.29, p = 0.006). In thrombolysed patients, both at baseline and after 24 h, LT and TTPeak were inversely correlated with TF, FXIa and FXIa, while Peak and ETP were positively correlated with those factors (data not shown).

**Table 1:** Baseline characteristics of stroke patients

Variable	All patients n = 95	Thrombolytic treatment status		p value
		Treated (n = 71)	Nontreated (n = 24)	
Age, years	75.0 (67.0–83.0)	75.0 (67.0–83.0)	76.5 (68.5–81.0)	0.97
Male sex, n (%)	47.0 (49.5)	33.0 (46.5)	14.0 (58.3)	0.31
BMI, kg/m^2^	27.0 (24.1–31.0)	27.6 (24.5–30.5)	26.0 (23.5–31.3)	0.66
Current smoking, n (%)	13.0 (13.7)	10.0 (14.0)	3.0 (12.5)	1.0
History of smoking, n (%)	28.0 (29.5)	20.0 (28.2)	8.0 (33.3)	0.63
NIHSS on admission	6.0 (3.0–13.0)	6.0 (3.0–14.0)	5.0 (2.0–10.0)	0.11
NIHSS after 24 h	3.0 (1.0–11.0)	3.0 (1.0–11.0)	4.0 (2.0–10.5)	0.57
Time from stroke onset to hospital admission, h	2.3 (1.75–4.75)	2.0 (1.6–2.6)	10.1 (7.0–20.3)	<0.0001
Medical history				
Arterial hypertension, n (%)	68.0 (69.4)	50.0 (70.4)	18.0 (75.0)	0.67
Atrial fibrillation, n (%)	19.0 (20.0)	15.0 (21.1)	4.0 (16.7)	0.23
Diabetes mellitus, n (%)	23.0 (24.2)	15.0 (21.1)	8.0 (33.4)	0.24
Hypercholesterolemia, n (%)	29.0 (30.5)	23.0 (32.4)	6.0 (25.0)	0.5
Heart failure, n (%)	20.0 (21.0)	17.0 (23.9)	3.0 (12.5)	0.23
Coronary artery disease, n (%)	32.0 (33.7)	24.0 (33.8)	8.0 (33.4)	1.0
Previous myocardial infarct, n (%)	20.0 (21.0)	17.0 (23.9)	3.0 (12.5)	0.23
Previous ischemic stroke, n (%)	12.0 (12.6)	8.0 (11.3)	4.0 (16.7)	0.5
Peripheral artery disease, n (%)	7.0 (7.4)	3.0 (4.2)	4.0 (16.7)	0.07
Medications				
Aspirin, n (%)	32.0 (33.7)	23.0 (33.8)	9.0 (37.5)	0.65
ACEI, n (%)	43.0 (45.3)	31.0 (43.7)	12.0 (50.0)	0.59
β-blocker, n (%)	46.0 (48.4)	35.0 (49.3)	11.0 (45.8)	0.77
Statin, n (%)	34.0 (36.2)	25.0 (35.2)	9.0 (37.5)	0.88
Anticoagulants, n (%)	7.0 (7.4)	6.0 (8.5)	1.0 (4.2)	0.67
Laboratory results				
Fibrinogen, µmol/L	8.1 (7.4–9.8)	8.0 (7.5–9.6)	8.7 (7.1–10.0)	0.70
APTT, s	32.0 (29.2–35.1)	33.2 (29.7–35.1)	31.8 (29–33.9)	0.45
INR	1.03 (0.98–1.10)	1.04 (0.98–1.10)	1.01 (0.96–1.10)	0.08
WBC, 10^9^/L	7.5 (6.2–8.9)	7.4 (6.1–8.8)	8.2 (6.5–9.1)	0.40
Platelets, 10^9^/L	206.0 (160.0–262.0)	196.0 (156.0–255.0)	223.2 (181.5–263.0)	0.42
Creatinine, µmol/L	86.6 (70.7–97.2)	84.0 (66.3–107.0)	76.9 (61.9–97.2)	0.21
Glucose, mmol/L	6.9 (5.7–9.2)	6.9 (5.9–9.0)	6.8 (5.7–10.9)	0.84
Total cholesterol, mmol/L	4.4 (3.6–5.1)	4.4 (4.5–5.0)	4.3 (4.3–5.4)	0.42
C-reactive protein, nmol/L	21.0 (9.5–55.2)	18.1 (6.7–65.7)	29.5 (13.3–48.6)	0.72

Data shown as median (IQR), or number (percentage)

*ACEI* angiotensin-converting enzyme inhibitors, *APTT* activated partial thromboplastin time, *BMI* body mass index, *CRP* C- reactive protein, *INR* international normalized ratio, *NIHSS* National Institutes of Health Stroke Score, *WBC* white blood cells

After 24 h no fatalities were observed, and neurological status improved in thrombolysed patients [NIHSS, median 3 (1.0–11.0), p < 0.0001], while in non-thrombolysed patients the NIHSS scores remained unaltered [median 4 (2.0–10.5), p = 1.0].

### Thrombin generation kinetics

On admission, TG parameters were similar in both groups (Table 2). Baseline Peak and TTPeak were associated with APTT (r = −0.28, p = 0.005 and r = 0.25, p = 0.013, respectively). NIHSS at baseline correlated inversely solely with TTPeak among the TG parameters (r = −0.21, p = 0.04). No associations between TG parameters and demographics, time from stroke onset to hospital admission, and NIHSS scores were observed.

**Table 2:** Thrombin generation, tissue factor, factor XIa and IXa: the impact of thrombolytic therapy

Variable	Patients receiving thrombolysis			Nontreated patients		
	At baseline	After 24 h	p value	At baseline	After 24 h	p value
Thrombin generation						
Lag time, s	748 (444–1166)	1356 (900–2420) *	<0.0001	653 (444–995)	729 (548.5–1507.8) *	0.27
Time to peak thrombin generation, s	1185.0 (751.0–1831.0)	1656.3 (1204.0–2401.0)*	<0.0001	881.0 (710.0–1536.5)	1033.0 (710.0–1656.3)*	0.8
Peak thrombin concentration, nM	65.1 (46.0–101.0)	42.5 (27.0–77.3)*	0.004	86.52 (53.55–106.3)	81.7 (48.1–138.5)*	0.38
Endogenous thrombin potential, nM s	74354.4 (49825.0–98086.0)	63561.0 (28165.0–80695.1)*	<0.0001	77070.4 (57743.0–99607.5)	92945.3 (65774.4–111302.5)*	0.41
Coagulation factors						
TF detectable, n (%)	10 (14.1)	14 (19.7)	0.45	4 (16.7)	6 (25)	0.72
FIXa detectable, n (%)	11 (15.5)	10 (14.1)	1.00	3 (12.5)	3 (12.5)	0.68
FXIa detectable, n (%)	21 (29.6)	23 (32.4)	0.85	10 (41.7)	9 (37.5)	1.00
Any two studied factors detectable, n (%)	14 (19.7)	18 (25.4)	0.50	6 (25.0)	6 (25.0)	0.72
All three studied factors detectable, n (%)	2 (2.8)	5 (7.0)	0.45	1 (4.2)	3 (12.5)	0.62

*FIXa* activated factor IX, *FXIa* activated factor XI, *TF* tissue factor

*p < 0.05 thrombolysed vs non-thrombolysed patients assessed after 24 h. There were no significant differences in any of the studied parameters between the groups at baseline

After 24 h since admission, the treated group showed decreased TG, reflected by 81.3% longer LT, 39.8% higher TTPeak, 34.7% lower Peak and 14.5% lower ETP, whereas there were no alterations to TG variables in the control patients (Table 2). Comparison of TG parameters assessed after 24 h since admission showed that thrombolysed patients had longer LT (by 86.0%, p = 0.002), higher ТТPeak (by 60.4%, p = 0.0011), lower Peak (by 48.0%, p = 0.0019) and lower ETP (by 31.6%, p = 0.0014) compared with the control group.

### Tissue factor, factor IXa and XIa

On admission, TF and FIXa were detectable in 14 (14.7%) patients (maximum levels, 6.4 pM and 1000 pM, respectively). FXIa was found in 31 (32.6%) patients (maximum, 30 pM). After 24 h TF was detectable in 20 (21.0%) patients (maximum, 2.7 pM), FIXa in 13 (13.7%; maximum, 180 pM) and FXIa in 32 (33.7%) patients (maximum, 5.7 pM). Shorter APTT on admission was noted in patients with detectable FIXa [30.7 s (27.9–33.4) vs 33.1 s (29.7–35.2), p = 0.01] and those with FXIa [31.0 s (28.8–33.4) vs 33.6 s (29.9–35.6), p = 0.02]. All the studied coagulation factors were positively intercorrelated, both in thrombolysed and non-thrombolysed patients, at baseline and after 24 h (data not shown).

Thrombolysis did not influence the percentage of individuals with detectable levels of TF, FIXa and FXIa, as well as their concentrations in blood in any of the two groups, and there were no intergroup differences in the studied coagulation factors at both time-points (Table 2). NIHSS score was not associated with the presence of detectable TF, FIXa or FXIa levels.

**Fig. 1:**
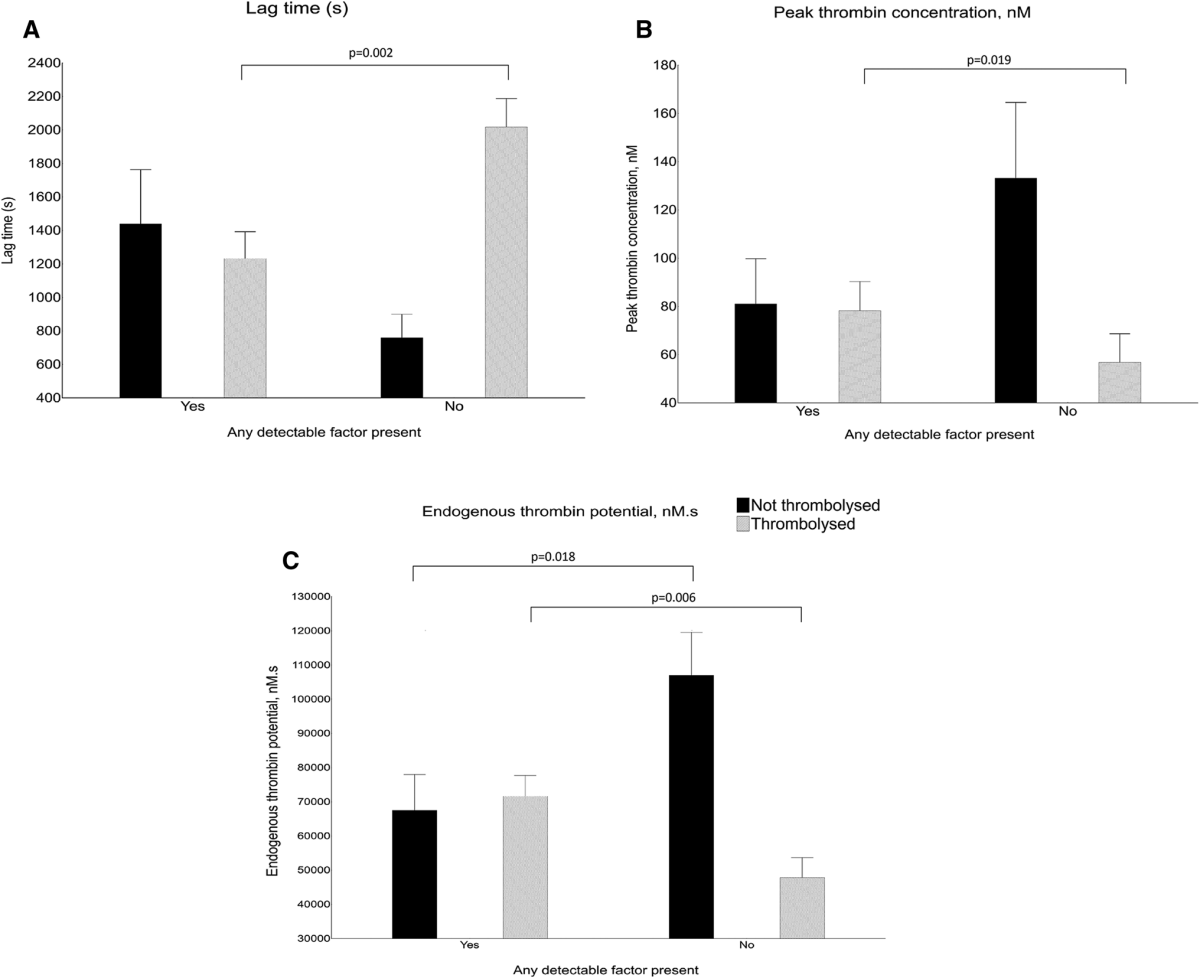
Comparison of patients with any detectable coagulation factor on admission with those without detectable coagulation factors: thrombin generation parameters measured after 24 h

Patients with a detectable level of any coagulation factor on admission were compared with those who did not have any circulating FXIa, FIXa and/or TF. In the nontreated group, TG parameters measured on admission were the same, and after 24 h patients with undetectable levels of the studied coagulation factors (n = 59) had higher ETP than those with detectable (n = 36, Fig. 1). However, among patients treated with thrombolysis, subjects with any detectable coagulation factor, as compared to the remainder, had enhanced TG, both at baseline [lower LT (both, p < 0.0001), shorter TTPeak (both p < 0.0001), higher Peak (both p < 0.0001), higher ETP (both p < 0.0001)] and after 24 h (Fig. 1.).

### High thrombin generation determinants

The multivariable analysis showed that FIXa measured at baseline was the only independent predictor for high ETP (the top quartile, >98934.0 nM s) in the whole studied group at baseline (Table 3) and the thrombolysis decreased the odds of high ETP by almost fivefold (the top quartile after 24 h, >93226.5 nM s). When FXIa was detectable after 24 h, the odds of high ETP were increased by more than threefold (Table 4). Moreover, in thrombolysed patients, the logistic regression showed that the only predictor of high ETP (the top quartile, >80695.1 nM s) was FXIa measured after 24 h (Online Resource 1). The multivariable model for the control group could not have been built.

**Table 3:** Multivariable logistic regression model for high endogenous thrombin potential (ETP) at baseline

Variable	ETP in the highest quartile at baseline (>98934.0 nM s)			
	Univariate analysis		Multivariate analysis	
	OR (95% CI)	p value	OR (95% CI)	p value
Age	1.01 (0.98–1.05)	0.78	1.00 (0.96–1.05)	0.88
Male sex	0.73 (0.28–1.87)	0.51	0.92 (0.30–2.85)	0.88
BMI	1.02 (0.92–1.12)	0.76	1.01 (0.90–1.13)	0.89
Fibrinogen	1.00 (1.00–1.08)	0.53	1.00 (0.99–1.01)	0.58
CRP	1.04 (1.00–1.08)	0.05	1.03 (0.98–1.08)	0.20
Previous ischemic stroke	2.58 (0.73–9.10)	0.15	–	–
Previous myocardial infarct	1.46 (0.49–4.39)	0.50	–	–
Atrial fibrillation	1.01 (0.29–3.48)	0.75	–	–
Diabetes mellitus	1.14 (0.39–3.36)	0.81	–	–
Previous smoking	1.39 (0.51–3.77)	0.53	–	–
Current smoking	0.93 (0.23–3.72)	0.92	–	–
Ischemic heart disease	1.75 (0.67–4.59)	0.26	–	–
Arterial hypertension	1.17 (0.40–3.37)	0.77	–	–
Internal carotid artery stenosis	1.17 (0.33–4.10)	0.81	–	–
LAA thrombus on TEE	1.53 (0.59–3.94)	0.38	–	–
Hypercholesterolemia	0.75 (0.26–2.16)	0.59	–	–
Aspirin	1.07 (0.40–2.87)	0.90	–	–
TF at baseline	2.85 (0.86–9.24)	0.09	2.13 (0.44–10.42)	0.35
FXIa at baseline	1.14 (0.42–3.06)	0.8	–	–
FIXa at baseline	5.78 (1.74–19.15)	0.04	6.25 (1.29–30.35)	0.02
Any studied factor detectable at baseline	2.77 (1.06–7.25)	0.04	0.96 (0.20–4.70)	0.96

The final model was adjusted for: age, sex, BMI, fibrinogen

*BMI* body mass index, *CRP* C- reactive protein, *FIXa* activated factor IX, *FXIa* activated factor XI, *LAA* left atrial appendage, *TEE* transesophageal echocardiography, *TF* tissue factor

**Table 4:** Multivariable logistic regression model for high endogenous thrombin potential (ETP) after 24 h

Variable	ETP in the highest quartile at 24 h (>93226.5 nM s)			
	Univariate analysis		Multivariate analysis	
	OR (95% CI)	p value	OR (95% CI)	p value
Age	1.00 (0.96–1.04)	0.96	0.99 (0.94–1.03)	0.55
Male sex	0.89 (0.34–2.33)	0.81	0.57 (0.16–2.04)	0.39
BMI	0.93 (0.85–1.04)	0.19	0.84 (0.82–1.07)	0.32
Fibrinogen	1.00 (1.00–1.00)	0.30	0.99 (0.99–1.01)	0.77
Previous stroke	3.75 (1.07–13.21)	0.04	2.74 (0.49–15.22)	0.25
Thrombolytic therapy	0.20 (0.07–0.58)	0.003	0.22 (0.06–0.78)	0.019
TF at baseline	1.86 (0.55–6.31)	0.32	–	–
TF after 24 h	2.01 (0.66–6.66)	0.21	–	–
FXIa at baseline	0.70 (0.24–2.04)	0.52	–	–
FXIa after 24 h	3.13 (1.14–8.56)	0.03	3.68 (1.10-12.27)	0.03
FIXa at baseline	1.58 (0.43–5.88)	0.49	–	–
FIXa after 24 h	2.22 (0.56–8.75)	0.25	–	–

The final model was adjusted for: age, sex, BMI, fibrinogen

*BMI* body mass index, *FIXa* activated factor IX, *FXIa* activated factor XI, *TF* tissue factor

## Discussion

This is the first study to demonstrate that the treatment of AIS with rtPA decreases TG with the strongest impact on LT. Of note, we observed that FIXa might identify patients with the highest TG initially after stroke, and high TG measured after 24 h is predicted by the presence of detectable FXIa levels in circulating blood. Thrombolysis did not influence the levels of TF, FIXa and FXIa and fractions of patients with detectable levels of circulating active TF, FIXa (both, approximately 20%) and FXIa (approximately 30%).

We used a modified assay based on the classic CAT introduced by Hemker [21], and this approach was successfully used by our group in several disease states [22, 23]. In contrast to TAT, our assay measures the maximum thrombin amounts that could be formed following activation by endogenous coagulation proteins like TF, FXIa and FIXa and phospholipids, therefore differences in the pattern of changes observed in the present study and the previous one by Tanne [5] are not surprising. Lack of differences in TG values measured within 72 h, and then 2 weeks after rtPA treatment in AIS patients in the study by Balogun et al. may suggest that the initial decrease in TG profile is transient [6]. However, one might speculate that decreased TG following thrombolysis at this early stage after AIS (within 24 h) has the greatest clinical impact.

Importantly, we observed that all TG parameters changed following rtPA treatment. Lower TG following thrombolysis might potentially produce additional beneficial effects beyond reduced prothrombotic action, since thrombin has been shown to be a proinflammatory, mitogenic mediator, and a trigger of neuron apoptosis [24].

We demonstrated active TF in 14.7% of patients on admission, and in 21% after 24 h—fractions lower than in our previous report, where active TF measured after 72 h since AIS onset was detectable in 33.1% of patients [25]. It indicates that active TF circulates less frequently in AIS within the first hours and increases with time in non-thrombolysed individuals. In the studied population with AIS and the intact blood-brain-barrier, the influence of the brain source of TF on plasma TF levels and thus also on TG is rather unlikely [26]. We demonstrated that a detectable level of FIXa on admission predicts the high TG in AIS. It has been shown that activation of FX by the intrinsic FXase complex (FIXa:FVIIIa), as compared to that by the extrinsic FXase complex (FVIIa:TF), is approximately 50-fold more efficient, thus making FIXa a potential marker for hypercoagulability, which could have practical implications.

Circulating FXIa is known as a marker of worse neurological outcome in AIS [25]. After 24 h, both in the whole cohort and in the rtPA treated group, FXIa predicted enhanced TG. This suggests that FXIa might be a marker of the stroke recurrence risk. Increased thrombotic risk in many disease states [14] known to confer with high FXI concentrations may be explained by an increase in ETP [27], which is consistent with this study, and/or impaired fibrinolysis and increased clot stability thus lessening the removal of thrombi. Nevertheless, this issue needs further investigations. It is worth mentioning that LT of TG is directly determined by the concentration of TF, FIXa, FXIa—by any of them individually, or any combination of the three, which is in line with our study results.

This study has several limitations. The size of the study group was limited but representative for real-life AIS patients. We did not study other coagulation factors e.g. prothrombin, however their impact on TG was documented previously. Potential factors influencing TG, e.g. fibrin degradation products, have not been measured. Although the used tests have very low threshold levels to detect TF, FIXa and FXIa, the undetectable levels did not mean a complete lack of activity. A prognostic value of the TG profiles following thrombolysis during long-term follow-up should be explored.

In conclusion, rtPA administered within 4.5 h of stroke symptoms onset attenuates the TG in AIS, and the FIXa test might identify patients with the highest TG initially after stroke. This study increases our knowledge on thrombolysis-induced changes in blood coagulation and supports the notion that clinicians should aim to implement rtPA in every eligible ischemic stroke patient as soon as possible.

## Electronic supplementary material

Below is the link to the electronic supplementary material.

Supplementary material 1 (DOCX 13 KB)
