# Supplementary material for: Activation of blood coagulation and thrombin generation in acute ischemic stroke treated with rtPA
Source: J Thromb Thrombolysis. 2017 Sep 6;44(3):362–70. doi: 10.1007/s11239-017-1544-7 (PMC5617868; doi:10.1007/s11239-017-1544-7)
Supplement: Supplementary file 1 — Supplementary material 1 (DOCX 13 KB) [file 11239_2017_1544_MOESM1_ESM.docx]

Table III. Multivariable logistic regression model for high endogenous thrombin potential (ETP) after 24 hours in thrombolysed patients.

| Variable | ETP in the highest quartile at 24 hours (>80695.1 nM.s) | | | |
| --- | --- | --- | --- | --- |
|  | Univariate analysis | | Multivariate analysis | |
|  | OR (95% CI) | p-value | OR (95% CI) | p-value |
| Age | 0.98 (0.94-1.03) | 0.42 | 0.99 (0.92-1.02) | 0.26 |
| Male sex | 0.79 (0.23-2.78) | 0.71 | 0.61 (0.11-3.26) | 0.56 |
| BMI | 0.86 (0.73-1.00) | 0.04 | 0.92 (0.78-1.10) | 0.37 |
| Fibrinogen | 1.00 (0.99-1.01) | 0.58 | 1.00 (0.99-1.01) | 0.86 |
| Previous smoking | 4.95 (1.35-18.22) | 0.02 | 4.79 (0.89-25.72) | 0.07 |
| TF after 24 hours | 2.45 (0.62-9.73) | 0.22 | - | - |
| FXIa at baseline | 1.92 (0.53-6.93) | 0.33 | - | - |
| FXIa after 24 hours | 3.76 (1.04-13.58) | 0.04 | 6.99 (1.06-46.00) | 0.04 |
| FIXa after 24 hours | 4.42 (1.02-19.16) | 0.06 | 2.83 (0.40-19.88) | 0.30 |

The final model was adjusted for: age, sex, BMI, fibrinogen. BMI, fibrinogen. BMI, body mass index; FIXa, activated factor IX; FXIa, activated factor XI; TF, tissue factor
